# What are the implications of implementation science for medical education?

**DOI:** 10.3402/meo.v20.27003

**Published:** 2015-04-23

**Authors:** David W. Price, Dianne P. Wagner, N. Kevin Krane, Steven C. Rougas, Nancy R. Lowitt, Regina S. Offodile, L. Jane Easdown, Mark A. W. Andrews, Charles M. Kodner, Monica Lypson, Barbara E. Barnes

**Affiliations:** 1 American Board of Medical Specialties Research and Education Foundation, Chicago, IL, USA; 2 The Colorado Permanente Medical Group, Denver, CO, USA; 3 Department of Family Medicine, University of Colorado School of Medicine, Denver, CO, USA; 4 Department of Medicine, College of Human Medicine, Michigan State University, Lansing, MI, USA; 5 Department of Medicine, Tulane University School of Medicine, New Orleans, LA, USA; 6 Department of Emergency Medicine, Alpert Medical School of Brown University, Providence, RI, USA; 7 Department of Medicine, University of Maryland School of Medicine, Baltimore, MD, USA; 8 Pamela C. Williams Clinical Skills and Medical Simulation Center, Meharry Medical College, Nashville, TN, USA; 9 Division of Clinical Skills and Competencies, Department of Medical Education, Meharry Medical College, Nashville, TN, USA; 10 Department of Anesthesiology, Vanderbilt University Medical Center, Nashville, TN, USA; 11 Department of Preclinical Medical Education, The Lake Erie College of Osteopathic Medicine at Seton Hill, Greensburg, PA, USA; 12 Department of Family and Geriatric Medicine, University of Louisville School of Medicine, Louisville, KY, USA; 13 Department of Internal Medicine, University of Michigan School of Medicine, Ann Arbor, MI, USA; 14 Graduate Medical Education, University of Michigan School of Medicine, Ann Arbor, MI, USA; 15 Continuing Medical Education, University of Pittsburgh School of Medicine, Pittsburgh, PA, USA; 16 Continuing Education and Industry Support, University of Pittsburgh School of Medicine, Pittsburgh, PA, USA

**Keywords:** dissemination and implementation, evidence translation, educational continuum

## Abstract

**Background:**

Derived from multiple disciplines and established in industries outside of medicine, Implementation Science (IS) seeks to move evidence-based approaches into widespread use to enable improved outcomes to be realized as quickly as possible by as many as possible.

**Methods:**

This review highlights selected IS theories and models, chosen based on the experience of the authors, that could be used to plan and deliver medical education activities to help learners better implement and sustain new knowledge and skills in their work settings.

**Results:**

IS models, theories and approaches can help medical educators promote and determine their success in achieving desired learner outcomes. We discuss the importance of incorporating IS into the training of individuals, teams, and organizations, and employing IS across the medical education continuum. Challenges and specific strategies for the application of IS in educational settings are also discussed.

**Conclusions:**

Utilizing IS in medical education can help us better achieve changes in competence, performance, and patient outcomes. IS should be incorporated into curricula across disciplines and across the continuum of medical education to facilitate implementation of learning. Educators should start by selecting, applying, and evaluating the teaching and patient care impact one or two IS strategies in their work.

1 July, 7:30 am.

One of several newly-formed patient care teams is assembling to begin morning rounds. Dr. Lin, a newly-minted supervising physician, will be leading rounds for the first time. Dr. Rice, a second-year resident, and Dr. Martinez, a new intern, check the patient roster. Two medical students arrive. A new pharmacy student and a new nursing student join the team. The nursing student notices that not everyone washes their hands before starting rounds and thinks about how much trouble she would get in to if her supervisor saw her forget to do that.

The team members introduce themselves to each other. Dr. Lin mentions that the hospital was found to be out of compliance with surgical site infection rates. Teaching services have been targeted for their potential to impact a large number of patients. Dr. Lin has volunteered to serve on a committee to propose a plan for bringing the hospital into compliance. Dr. Rice asks: ‘What is the “differential diagnosis” of having too many infections in a hospital? How do you figure that out and fix it?’

Implementation science (IS) utilizes theories, models, principles, and methods derived from multiple disciplines and industries outside of medicine (e.g., organizational development, quality improvement, industrial engineering, business management) to ‘promote the systematic uptake of research findings and other evidence-based practices into routine practice … to improve the quality and effectiveness of health services’ (1). By providing frameworks for translating and implementing new knowledge and skills in work settings, IS can help medical educators promote and measure behavior change in their learners. The integration of IS training across the spectrum of medical education can also provide assist in achieving milestones and competencies assessed by major accrediting agencies such as the Accreditation Council on Graduate Medical Education (ACGME) in their ‘Next Accreditation System (NAS) and Clinical Learning Environment Reviews (CLER)’ (2) the Accreditation Council on Continuing Medical Education (ACCME) (3), the American Board of Medical Specialties (ABMS) in their Maintenance of Certification (MOC) program, (4) the Royal College of Physicians and Surgeons of Canada, and the General Medical Council in the United Kingdom (5, 6).

This review highlights selected IS theories and models, chosen based on the experience of the authors, that could be used in planning and delivering medical education activities to help learners better implement and sustain effective education in their work settings. We discuss the necessity of using IS approaches for education at the level of the individual, team, and organization, as well as its integration across the medical education continuum from undergraduate and graduate training through continuing professional development. We also discuss the importance of determining whether IS enhances learning and leads to improved outcomes of care. We briefly identify potential barriers to incorporation of IS into medical training and identify potential next steps to implementation. Finally, we provide lists of Key Points and Strategies for moving this work forward.

## Selected IS theories, models, and tools relevant to medical education

Most practicing physicians should be provided with tools and practical techniques to use in their work to identify and solve practice-based problems, work effectively with their teams, translate new knowledge and skills into practice, and implement continuous practice improvement in their organizational context. While we contend that medical educators will need firm foundational underpinnings in aspects of IS, most will not need to become ‘implementation scientists’ *per se*. However, medical educators need to understand and incorporate several different IS approaches in their educational design and delivery. This might mean working with others (quality improvement, clinical systems leadership) to identify and prioritize gaps in practice based on local contexts, anticipating or identifying barriers to implementation of learning (7) and proactively addressing them as part of educational activities, and providing IS-based tools to facilitate learner practice change. While an exhaustive review of IS is beyond the scope of this paper, a few approaches pertinent to medical education at the level of the individual, team, and within organizational contexts are discussed.

## IS to target change in individuals

Educational interventions are intended to improve the knowledge, competence, and/or performance of individual learners. Table 1 highlights selected theories and models used in IS that are applicable at the level of the individual learner and gives examples of how they can be utilized in educational activities. Curriculum designers and educational researchers could collaborate to study the effect of incorporating these theories into educational activities on desired learner outcomes.

**Table 1:** Selected change theories models used in implementation science applicable to medical education at the level of the individual learner

Theory/model	Relevance to medical education	Example
Theory of planned behavior/reasoned action (8)	Use to help prepare individual learners for change and applying new learning. These theories posit that intent to change behavior precedes actual change. Intent to change is influenced by attitudes, beliefs, motivation, subjective norms (of peers and respected others), and perceived control of a situation.	In order to improve hand washing behavior, educational interventions need to convince an individual to regularly wash their hands. This could be done using social influences (highly regarded peers), showing the connection between lack of hand washing and infection rates, providing motivation (stories of success) and easily accessible reminders to wash hands.
Prochaska and DiClemente’s Transtheoretical model (9)	Facilitate individual behavior change, by using different educational foci and strategies for those who are pre-contemplative (unaware that change is needed), contemplative (considering a behavior change), preparing to make a change, and those who have already made a change and need to maintain or improve their efforts.	Individuals may be unaware of the linkage between lack of hand washing and nosocomial infection. Once made aware and contemplating a change, the pros and cons of routine hand washing can be discussed, with strategies to support the behavior and preparation for potential downsides (e.g., use of moisturizer to prevent dry skin). Discussion of successful peer practices can help those preparing to make a change and those who have started to more regularly wash hands but occasionally forget.
The PRECEED/PROCEED model (10)	Address predisposing (e.g., attitudes, beliefs, previous experience), reinforcing (e.g., follow-up with audit and feedback or ongoing support) and enabling (e.g., algorithms, decision support) factors to change and learning implementation. Has been shown to facilitate practice change when specifically applied in continuing professional development (11).	Barriers to hand washing may include predisposing attitudes or beliefs (it’s not important; it’s a waste of time). Enabling factors could include signs on the hospital room door or at the bedside. Reinforcing factors could include random audits and a ‘prize for most times caught hand washing’.
The Pathman model (12, 13)	Tailors education so that individuals are aware of information and need for change, agree with the importance of change, helps them adopt practice changes, and adhere to practice changes over time.	Provide information so individuals are aware of the evidence and rationale behind hand washing (aware), engage them in discussion to surface concerns or doubt to help convince them to change their behavior (agree), provide support (reminders, visual prompts) to adopt the change, and reinforce adherence to hand washing over time.
Using principles of cognitive psychology to aid in learning transfer (14)	Giving a principle with multiple examples increases the chance of learning transfer into practice compared with giving the principle alone or with a single example.	Embedding multiple case examples from the literature linking hand washing with surgical site or other nosocomial infections and demonstrating successful hand washing reminder and implementation efforts (or having participants identify these methods).
Force field analysis (15)	Use with individuals or groups to look at positives and negatives of a situation. Can be used to help convince learners that change is needed.	Using diagrams to connect the evidence behind hand washing and infection rates and showing how barriers can be practically overcome.

## IS targeting teams

The nursing student listens to the ward team’s conversation. She doesn’t understand all the terminology, but she does understand how things happen on the floor and sometimes is surprised by the way things are done or not done. Hand washing seems simple enough. Why doesn’t everyone here do it? She has some ideas about how that could be changed, but is hesitant to raise the issue.

Whether in large organizations or small practices, health care is increasingly being delivered by interprofessional teams. Meaningful change (especially complex change) that results in improved health outcomes depends more on team improvement than changing individual physicians. As reflected by the opening vignette, our current health care system lacks both collaborative practice across health professions and a shared understanding of what this means. Team-based care is needed to meet competencies outlined in several frameworks: the Institute of Medicine (16), CanMeds (17), and the Scottish Doctor (18). Because medical professionals learn primarily in silos, they are often ill equipped to work together to meet the needs of their patients and unprepared to function in teams. Reports by the Josiah Macy, Jr., Foundation (19), the Interprofessional Education Collaborative Expert Panel (20), and the European Interprofessional Education Network (21) call for changes in medical education that emphasize interprofessional, team-based care, and community-based education for the entire continuum of medical education. Educators should develop educational interventions to help individuals function better as team members and help teams grow and improve together. Until recently, training on ‘how to be a team player’ has not been a routine part of medical education. Efforts are underway at the undergraduate health care level (22) and in graduate medical education (2) but are nascent for practicing professionals. The ACGME CLER visits specifically include assessments of faculty educational initiatives and faculty outcomes as a result of those new efforts (23).

For individuals to function as a team, and for teams in general to be effective, several key competencies (24, 25) should be developed (Table 2). A basic knowledge of team development (i.e., ‘Form, Storm, Norm, Perform’) (26) could help educators tailor educational activities to the team’s stage of function.

**Table 2:** Key team-based competencies for medical educators to target in their learners

Interdisciplinary communication (with attention to avoiding jargon that is not meaningful across disciplines)
Role clarity and knowledge, so that each member of the team knows what other team members are responsible for in a given task
Respectful communication to ensure clarity as well as psychological safety to ensure that concerns can be raised without fear of implicit or explicit retribution or criticism
Development/negotiation of shared (team) goals
Change management/adaptability/conflict resolution
Shared leadership and decision making clarifying approaches of how to decrease hierarchical care when appropriate
Values/ethics for team-based practice
Patient-centered care as the focus compared to the focus on the professional or systems
Mutual performance monitoring and accountability (team members providing feedback to each other and holding each other accountable to the team for performance)
Handoffs (including ‘practical know-how’ on sharing patient care so that care transitions occur smoothly from the patient perspective and critical information and follow-up items are communicated between professionals in different care settings)
Cooperation (working to achieve shared goals) and collaboration (working together to generate new approaches)
Shared workload

Derived from: Baker et al. (24); Sargeant et al. (25); Interprofessional Education Collaborative Expert Panel (20).

## IS targeting organizations

Dr. Lin considers the daunting task of providing appropriate oversight and teaching for her diverse team. She starts the morning by describing her training trajectory and disclosing that this is her first month in a new role. She asks the resident, the intern, the medical students, the nursing, and pharmacy students to describe their role, their expectations, their last rotation, a recent success, and their worst fear about this upcoming month. Everyone wonders if it is actually ‘safe’ to talk about their worst fears. Slowly, they begin to share with each other. The senior resident has never organized work rounds before. The intern feels ‘rusty’, having not done inpatient care for several months after less intense electives and vacation. The medical students need to hand in 10 patient write-ups that demonstrate applications of best evidence to patient management plans; they are not sure they know what that means. They also wonder who ‘all these people in different colored scrubs are’. The pharmacy student is not sure who they will be working with – the senior resident? The intern? The nurse? What is a nurse intern?

The first day is filled with patient care activities. That evening, Dr. Lin attends the first meeting of the committee that will work towards surgical site infection reduction. It feels like an unwieldy problem.

The commonly used educational planning sequence (problem identification/gap analysis, targeted needs assessment, determination of objectives, selection and delivery of appropriate educational formats, evaluation, and follow-up) (27) align with several implementation strategies such as the Plan–Do–Study–Act cycle (28), Rogers’ Diffusion of Innovations (29), and Complex Adaptive Systems Theory (30, 31). A previous publication (32) describes how these models can be proactively used in developing educational interventions. In addition, Six Sigma (33) and Lean Thinking (34) can be applied to develop educational interventions to improve patient safety and promote efficiency in the delivery of health care. Examples exist utilizing a SOAP note format to guide clinicians accustomed to using this approach in patient care toward a broader, system-level view of needs for educational improvement (35). Table 3 shows how some of these constructs can be applied by the hospital to address the problem of surgical site infections in the vignette.

**Table 3:** Examples of implementation science models used within organizations

Construct	Example
Complex adaptive systems theory (30, 31)	Hospital administration identifies teams to pilot new surgical site infection reduction initiatives (such as Dr. Lin in the vignette) based upon her dissatisfaction with current infection rates, agreement that changes are needed, and willingness to involve her team to try new things to affect improvement.
Plan–do–study–act cycle (28)	Several patient care teams develop a test of change to promote hand washing behavior. Care team in vignette does the following: *Plan* – team places hand washing posters over all of the patient beds on one floor, and asks patients (when able) to remind all hospital staff to wash hands before touching the patient. *Do* – Plan is carried out for 1 week. *Study* – Team self-reports hand washing (yes or no) after each patient encounter on a paper form taped to all electronic health record terminals on the floor. After 1 week, team studies the results, and asks patients if they mind providing such reminders. *Act* – Team notes that most patients are reluctant to remind staff to wash hands, and that self-reporting data collection was incomplete. Posters were highly visible and generally felt to be helpful. Team meets to identify different methods to more accurately track hand washing.
Six Sigma (33, 34)	The senior resident decides to study the use of Six Sigma principles to decrease surgical site infections as a residency research project, and asks Dr. Lin to act as mentor. Define: Dr. Lin’s committee defines the goal and scope of their initiative: ‘Decrease surgical site infections experienced by patients on 5 South by 25% over the next 3 months’. Measure: Surgical site infections suffered by 5 South patients for prior 6 months is performance baseline. Analyze: Dr. Lin and Dr. Rice develop a plan to work with IT within the hospital to create monthly reports on 5S patient surgical site infection outcomes. Improve: Root cause analysis teams will evaluate 3 surgical site infection cases from the previous 6 months and 1 case each (if there is an occurrence) over each of the next 3 months study period. Control: ongoing statistical analysis will monitor process and outcomes.
Diffusion of innovations (29)	Surgical site infection reduction methods are selected for wider spread based on: *Simplicity* of the new workflow *Observability* – ability to watch other individuals implement new workflows. *Relative advantage* of the new workflows compared to previous workflows *Trialability* – ability for individuals and teams to try the workflow and adapt it to their practice. *Compatibility* of the new workflows and tasks with other job responsibilities.

## IS tools

IS uses tools from many fields and disciplines – a few that seem to hold promise for use in medical education include structured approaches to identifying and analyzing problems, methods of implementing evidence in the context of the team and organizational environment, and analytic techniques that evaluate and display changes over time (36–39). Table 4 shows some of these tools, their application to medical education and examples of how they could be applied in the situation outlined in the vignette. Many of these tools can be used in mixed-methods evaluation of impact; qualitative methods in particular may be useful in identifying key factors that can assist in the translation of improvement between different contexts and settings.

**Table 4:** Examples of implementation science tools of potential use by medical educators

Tool	Potential use in medical education	Example
The ‘success factor profile^©^’ (36), a series of 5 items with an 8-point Likert scale rating of the following descriptors of success likelihood with new medical information technology: Past success with innovation/implementation Generalizability of new skill/learning Leadership and enthusiasm to embrace change Learning opportunities for development and implementation teams to improve Subjective rating of probability of success	Predict the best sites to introduce new medical information technology or educational activity focused on other types of new innovation Identify early adopters for ‘train-the-trainer’ educational interventions, who can then show others how to make and sustain changes in practice	The senior resident has used the EMR to extract data for conferences in the past. Using the EMR to develop a form for use in his surgical site infection project seems promising Dr. Lin’s team decides they ‘want to be a part of the solution to the high surgical site infection rates rather than a part of the problem’. They offer to help the senior resident with his project
Histograms, control charts, run charts, interrelationship digraphs and Pareto charts (37), and Fishbone diagrams (38)	Identify priority areas to focus on in an educational activity Illustrate gaps between current and desired performance Illustrate best practices	A Fishbone diagram is developed to illustrate possible contributing factors to surgical site infections. The team uses this to identify potential practice gaps and focus areas for the infection reduction effort A control chart of surgical site infections is posted in the care team gathering area. This shows the month’s goal rate, the rate of the best comparable hospital in the country, and progress toward goals over time
Six Sigma concepts (33) focuses on developing and implementing consistent, efficient, reliable (error-free) processes to predictably meet the needs of a customer from the customer’s frame of reference	Identify the needs and desires of the users (clinicians, other staff), stakeholders (organizational leaders), and beneficiaries (patients) of an educational activity Identify desired educational and learning implementation steps Develop ‘performance-improvement’ educational activities and maintenance of certification part IV activities (American Board of Medical Specialties, 2015)	The needs assessment identifies that multiple stakeholders in the process think a checklist of steps would help, as would the ability to practice the steps together A simulation scenario is developed using standardized patients and partial task trainers and both residents and attending physicians accrue recognition and CME credit for participating. Three flawless performances and a re-demonstration of flawless performance every 3 months are effective in maintaining skills in 92% of participants
Team STEPPS^®^ system (40), a multimedia toolkit providing pre-training assessment, onsite training guide, and support for implementation and sustaining of new team behaviors focused on patient safety.	Address multiple team competencies in an interprofessional education setting to improve patient safety	Team members provide ongoing support for each other and utilize huddles, briefing and debriefing strategies routinely as they care for patients
The American Association of Medical College’s Teaching For Quality (Te4Q) initiative (22, 41), a set of recommendations, competency and evaluation frameworks, to guide faculty development in quality improvement principles.	Resources for use in faculty development courses to assist medical educators with using IS approaches in medical education	The team aligns their activities and goals with the Te4Q competency framework which enables the senior resident to demonstrate aspects of practice-based learning and improvement (PBL&I) requirements to the residency director and aids Dr. Lin in labeling and prioritizing the activities of the ad hoc committee

## Incorporating IS along the continuum of medical education

Undergraduate and graduate medical training programs increasingly are required to develop curriculum and assessment metrics appropriate for the clinical learning environment (from academic medical center to community practice settings) in which trainees receive both the knowledge and experience of skill acquisition and meet specified milestones in these domains. ACGME CLER visits, for example, seek to specifically assess that environment on six axes (patient safety; health care quality and reduction in health care disparities; transitions in care; supervision; duty hours and fatigue management and mitigation; and professionalism) that reflect the culture of learning and patient care (23). Active learning and continuous application of IS can assist in the achievement of these competencies and outcomes, to help students and residents transition to real-world practice prepared to make meaningful contributions to improve the processes and outcomes of care delivery. IS strategies can also help faculty adjust to the more safety and quality-focused expectations of both accreditation and credentialing/MOC bodies.

Introducing this material at the start of the undergraduate curriculum requires flexibility of approach but as shown with other new curricula, can be accomplished successfully. For example, the inclusion of medical error scenarios into a series of patient cases in a problem-based learning curriculum resulted in demonstrable improvement in learner knowledge of medical error genesis, failure modes, and root cause analysis principles (42). Medical educators have devised ways to integrate other important themes, such as communication skills, cultural competence, and ethics into existing curricula. IS presents a rich educational opportunity – learners who understand IS principles can begin to utilize those principles to address both educational and clinical challenges in a unique and reinforcing way. Similarly, thoughtful approaches are needed to incorporate IS into continuing medical education activities for practicing physicians, to better equip them to work with their teams to care for populations of patients with increasingly complex and multifaceted problems, while attending to and improving preventive services. Accrediting, licensing, and credentialing bodies are calling for the creation of explicit goals and objectives for educational programs across the continuum and the provision of explicit pathways to competence so that learners demonstrate the ability to work in interdisciplinary teams, employ evidence-based practices, apply quality improvement concepts, and use high-quality medical informatics (43). Table 5 suggests IS competencies that the literature and the authors’ experience suggest are relevant to different learners and learner groups from trainees to experienced practitioners and from the individual through the team to the organization.

**Table 5:** Implementation science milestones relevant to different learners and learner groups

Learner or learner group	IS competency examples
Students	Identifies personal strengths and weaknesses and develops ongoing personal learning plans Demonstrates receptiveness to faculty and peer/colleague feedback as a means of facilitating personal and professional improvement Locates, appraises, and assimilates evidence from scientific studies related to their patients’ health problems Demonstrates respect for all members of the health care team Demonstrates understanding of the principles of, and functions as a member of a fail-safe team Demonstrates knowledge of differing types of medical practice and delivery systems and their implications for controlling health care allocation and cost
Residents/advanced training/supervised practice	Identify strengths, deficiencies, and limits in one’s knowledge and expertise Set learning and improvement goals Identify and perform appropriate learning activities Systematically analyze practice using quality improvement methods and implement changes with the goal of practice improvement Incorporate formative evaluation feedback into daily practice Locate, appraise, and assimilate evidence from scientific studies related to their patients’ health problems Use information technology to optimize learning; participate in the education of patients, families, students, residents, and other health professionals
Independent practitioners	Practice-based learning and improvement – able to investigate and evaluate their patient care practices, appraise, and assimilate scientific evidence and improve their practice of medicine Systems-based practice – demonstrate awareness of and responsibility to larger context and systems of healthcare. Be able to call on system resources to provide optimal care (e.g., coordinating care across sites or serving as the primary case manager when care involves multiple specialties, professions or sites) Apply quality improvement – identify errors and hazards in care; implement basic safety design principles; continually measure quality of care in terms of structure, process and outcomes; design and test interventions to change processes and systems of care Utilize informatics – communicate, manage knowledge, mitigate error, and support decision making using information technology Describe one’s roles and responsibilities clearly to other professions Recognize and observe the constraints of one’s role, responsibilities, and competence, yet perceive needs in a wider framework Recognize and respect the roles, responsibilities, and competence of other professions in relation to one’s own Work with other professions to effect change and resolve conflict in the provision of care and treatment Work with others to assess, plan, provide, and review care for individual patients Tolerate differences, misunderstandings, and shortcomings in other professions Facilitate interprofessional case conferences, team meetings, etc. Enter into interdependent relations with other professions
Teams	Demonstrate respect for all members of team Avoid intimidation in team interactions Use closed-loop communication Plan care activities to maximize participation of all team members Use brief and debrief activities Avoid jargon
Organizations	Specify IS competence/training for high reliability as goal of institution/organization Incorporate IS use into reward system Require performance assessment of team competencies

Derived from: Barr (44); Institute of Medicine (16); Sousa et al. (45); ACGME (46); Interprofessional Education Collaborative Expert Panel (20); American Board of Medical Specialties (4).

## Community-based teaching settings

Students, residents, and other healthcare professionals are and will be increasingly learning in non-hospital practice settings (47–50). These sites constitute the overwhelming bulk of the medical landscape (51); trainees will experience variability in the adoption and implementation of evidence-based and quality/outcomes-based practices in these more dispersed and de-centralized ‘real-world’ settings. Practicing physicians are required to evaluate their own practice and demonstrate evidence of continuous learning and improvement, ensuring that they are appropriately implementing the latest scientific principles to deliver safe, quality care, and improve patient outcomes (4). Similar to institutionally-based medical educators, most community faculty will not need to become broad IS subject matter experts. However, they will be required to incorporate learnings about IS in their daily work, enabling them to critically evaluate and improve the care they provide, and using them in their MOC/Competence efforts. This will equip them to model and teach these approaches to their learners (52).

At rounds the next morning, Dr. Lin describes the surgical site infection committee meeting. All agree that it will be a challenge to figure out why surgical site infection rates are high and how to lower them. The senior resident wonders how many people touch a patient each shift, how to study that, and whether he can use this as a research project.

## Studying educational activities that incorporate IS

As described thus far, the theories and models of IS hold great promise for the evolution of medical education, but incorporating these approaches in our current system is no small undertaking. Educational and IS researchers must collaborate with educational activity planners to study whether adding IS approaches to education improves practitioner competence and performance, patient outcomes, and population health. While some of these changes can be assessed by self-report with commitment-to-change statements and barrier analysis assessed on immediate and follow-up education evaluation forms, we need additional outcomes-focused data to more fully assess educational outcomes.

Simulation-based training (including standardized patients) can be used to evaluate some competencies and individual and team performance changes in care processes after educational activities. Low-fidelity simulation (e.g., evolving patient scenarios) as well as high-fidelity simulation with mannequins and specialized venues may be useful, depending on the desired change and its complexity. McGaghie (53) and others have shown that simulation strategies can result in improvements that translate from the simulation laboratory to the bedside. These educational interventions require input from multiple educational ‘team’ members as well as ‘care team’ member input in their design and execution (53). However, simulation experiences (and application of other educational tools and constructs) under controlled and somewhat idealized circumstances may not always translate to the real world, thus underscoring the importance of follow-up assessment of effectiveness. Assessing the effect of implementation-focused, interprofessional team educational activities on care processes and outcomes and team functioning is also important, using or adapting existing team assessment tools or creating new validated instruments. Such tools could also identify gaps in team functioning that can be specifically addressed with follow-up professional development opportunities.

## Barriers to incorporating IS into medical education

Across the spectrum (from medical school clinical experiences to GME to community practice settings), faculty and learners must be provided with timely, accurate, and regular data in order to identify areas for improvement and assess the progress toward their goals. User-friendly registries for data extraction from electronic health records, medical claims, and other data systems need to be accessible for use by teachers and learners in both institutional and community settings. IS must be supported and prioritized by faculty and institutional leaders, and achievement of IS competency by faculty and students must be made explicit and financially supported goals. Trainees must see IS applied in the real world of patient care or it will be devalued. This re-emphasizes the need to weave IS into curricula across the professional spectrum so that newly trained practitioners understand it, and practitioners can realistically experience its value. It also provides the opportunity to involve learners at all levels in solving real-world challenges together as they learn together.

Established faculty, physicians, and other health care givers have a strong tendency to maintain their current routines. Initiating an educational push for best practice implementation in undergraduate medical education and maintaining this in graduate medical education and clinical practice is necessary. Institutionalizing this new orientation in undergraduate and graduate medical education settings will require several steps. Finding a motivated cohort of early adopter educators who will be willing to champion change and equipping them with knowledge and skills to foster change across the continuum of medical education will be very important. These early adopters will help convince and teach other educators how to teach and incorporate IS into their work; thus, they will need to be located in the workplace where teaching occurs. Securing appropriate time for educator development and curricular time (in the face of competing demands) to incorporate IS competencies and securing funding and economic factors, including an understanding that implementation of best practices, will initially have a cost in time and dollars (54, 55). Faculty development programs for physician educators must be developed, but this also means that these programs must be valued in terms of financial support and faculty promotion. Using IS strategies to help meet accreditation and credentialing requirements could help facilitate these changes in organizational culture.

Rounds are animated the next morning. Case presentations include some new questions: ‘How could this patient’s problem have been prevented?’ ‘What are the risks to this patient during their hospitalization?’ ‘How can the team keep track of what everyone is doing?’ ‘How can rounds better take advantage of the skills and perspectives of different team members caring for these patients?’ ‘How many hands DO touch a patient each day?’

## Conclusions and next steps

‘Knowledge is not enough – we must do’. – Goethe

The implicit goal in newer models of medical education that teach and incorporate IS is not just knowledge acquisition (which is necessary but not sufficient) but behavior change and improvement. One potential implication of these new benchmarks is the need to abandon potentially outmoded jargon. For example, while ‘learning objectives’ are supposed to be articulated in specific, actionable, behavioral, observable, implementable, and measurable terms, it is not uncommon for them to be teacher centric (‘what I am going to teach you’). Some now ask for ‘performance expectations’ instead of learning objectives, to explicitly focus attention on what we expect learners to do. Thinking beyond ‘what do we want learners to learn’ to ‘what’s the problem we’re trying to solve’ and ‘how will we know we’ve been successful in improving this problem’ will require a thoughtful integration of IS into medical education design and implementation. Imparting the knowledge, skills, and attitudes necessary to utilize IS into medical education holds promise for helping us better achieve changes in competence, performance, and patient outcomes; but we must be sure to thoroughly assess these changes to validate (or invalidate) these assumptions.

We believe that IS should be incorporated into curricula across the continuum (undergraduate, graduate, and continuous professional development level) to help provide tools and strategies for implementation of learning. All disciplines should ideally learn and use IS together, and the ability to use IS should be an explicit outcome of our teaching. We encourage educators, based on their local needs and priorities, to choose, apply, and evaluate one or two of the strategies outlined in this paper, working with others to assess their impact on teaching and on the outcomes of care.

20 May the following year.

Together, all members of the care team developed and refined a process and improved their adherence to a hand hygiene checklist. Hospital information technology placed this checklist into the electronic health record so that teams across the hospital can access and utilize it. Data is accumulating on the number of care teams accessing the checklist and their patient’s rates of surgical site infections, and systems have been developed to regularly provide this data to the team. Individually, each team member has tried different types of reminders to improve their personal rates of hand washing. They also participated in hospital-sponsored seminars on teamwork and communication. They have gradually become more inclusive in their communication styles and have developed a comfort speaking up when problems arise. Hospital administration has reviewed the data and has noted that patients cared for by these individuals seem to have lower nosocomial infection rates.

The team members have a good sense of each other’s strengths and weaknesses. Rounds have become more efficient as each team member knows what they are responsible for checking and reporting. As a team, they can often answer questions that might have waited for a different member to arrive, or they can quickly access the right member when specialized knowledge is necessary. When issues arise, they often use Plan–Do–Study–Act cycles to quickly make changes and determine their effectiveness. They have begun to think about solving problems in a fundamentally-different way.

The team is conducting pre-op bedside rounds on Mr. Davis, who is scheduled for a partial colon resection. The team proactively discusses ways to decrease Mr. Davis’ risk of post-op surgical site infection. Mr. Davis observes the collaborative nature of the discussion and is encouraged to ask questions. He enters his surgery impressed with and confident in the ability of his health care team.
